# Circular RNA and Its Roles in the Occurrence, Development, Diagnosis of Cancer

**DOI:** 10.3389/fonc.2022.845703

**Published:** 2022-04-07

**Authors:** Yue Zhang, Xinyi Zhang, Yumeng Xu, Shikun Fang, Ying Ji, Ling Lu, Wenrong Xu, Hui Qian, Zhao Feng Liang

**Affiliations:** ^1^ Jiangsu Key Laboratory of Medical Science and Laboratory Medicine, School of Medicine, Jiangsu University, Zhenjiang, China; ^2^ Child Healthcare Department, The Fourth Affiliated Hospital of Jiangsu University, Zhenjiang, China

**Keywords:** circRNAs, biogenesis, cancer, biomarkers, mechanisms

## Abstract

Circular RNAs (circRNAs) are non-coding single-stranded covalently closed circular RNA, mainly produced by reverse splicing of exons of precursor mRNAs (pre-mRNAs). The characteristics of high abundance, strong specificity, and good stability of circRNAs have been discovered. A large number of studies have reported its various functions and mechanisms in biological events, such as the occurrence and development of cancer. In this review, we focus on the classification, characterization, biogenesis, functions of circRNAs, and the latest advances in cancer research. The development of circRNAs as biomarkers in cancer diagnosis and treatment also provides new ideas for studying circRNAs research.

## 1 Introduction

CircRNAs were first discovered in RNA viruses in 1976 (1). Subsequently, it was discovered in eukaryotic cells and humans (2–4). CircRNAs are covalently closed ring structures with 5 ‘and 3’ ends directly linked together, which makes them more stable than linear RNA. They were originally thought to be the product of splicing errors during low abundance transcription. With the development of high-throughput RNA sequencing technology and bioinformatics algorithms, a new understanding of circRNAs has emerged. The functions and mechanisms of new types of circRNAs during biogenesis have been identified. CircRNAs have been found to act as ceRNA or miRNA sponges and bind to proteins. As well as some newly discovered functions, such as regulating parental gene expression, regulating pre-RNA splicing and potential translation templates for proteins (5). Many circular RNAs have been discovered to be biomarkers that impact the onset and growth of malignancies in recent years, attracting a lot of attention. They have been identified in lung cancer (6), hepatocellular carcinoma (7), gastric cancer (8), colorectal cancer (9), and so on. However, the molecular mechanisms and early diagnosis of cancer are not well understood. And, diagnosis and treatment based on circRNAs are still lacking. Therefore, it is urgent to explore new molecular mechanisms and effective biomarkers for the diagnosis of cancer.

In this review, we focused on the biological characteristics, functions, mechanisms, and detection techniques of circRNAs associated with cancer, and discussed their potential application as biomarkers and therapeutic targets. Thus, provide valuable clinical information for the diagnosis and timely treatment of cancer in the future.

## 2 CircRNAs

### 2.1 Classification and Properties of CircRNAs

According to the formed sequence, circRNAs can be divided into six categories: exonic circular RNAs (ecircRNAs), circular intronic RNAs (ciRNAs), exon-intron circular RNAs (EIciRNAs), intergenic circRNAs, anti-sense circRNAs, and tRNA intronic circRNAs (tricRNAs) (10).

CircRNAs have no ends so it is highly stable and have specific spatiotemporal expression patterns. Numerous circRNAs usually express in specific tissues and specific developmental stages (11). CircRNAs were found to be evolutionarily conserved in diversity and the conservation is different in different tissues, among which the most conservative in the brain (12). Jeck et al. identified over 25,000 different circRNAs in human fibroblasts (13). In addition, circRNAs are widely distributed and have been reported in thousands of animal cells, such as humans, mice, and nematodes, and expressed in high abundance (11, 14). Rybak-wolf et al. found that circRNAs are abnormally enriched in the mammalian brain and are specifically and dynamically expressed in neuronal differentiation (15). New classification methods such as differences in length, stability, function, and characteristics of circRNAs still need to be continuously explored.

### 2.2 Biogenesis of CircRNAs

**Figure 1:**
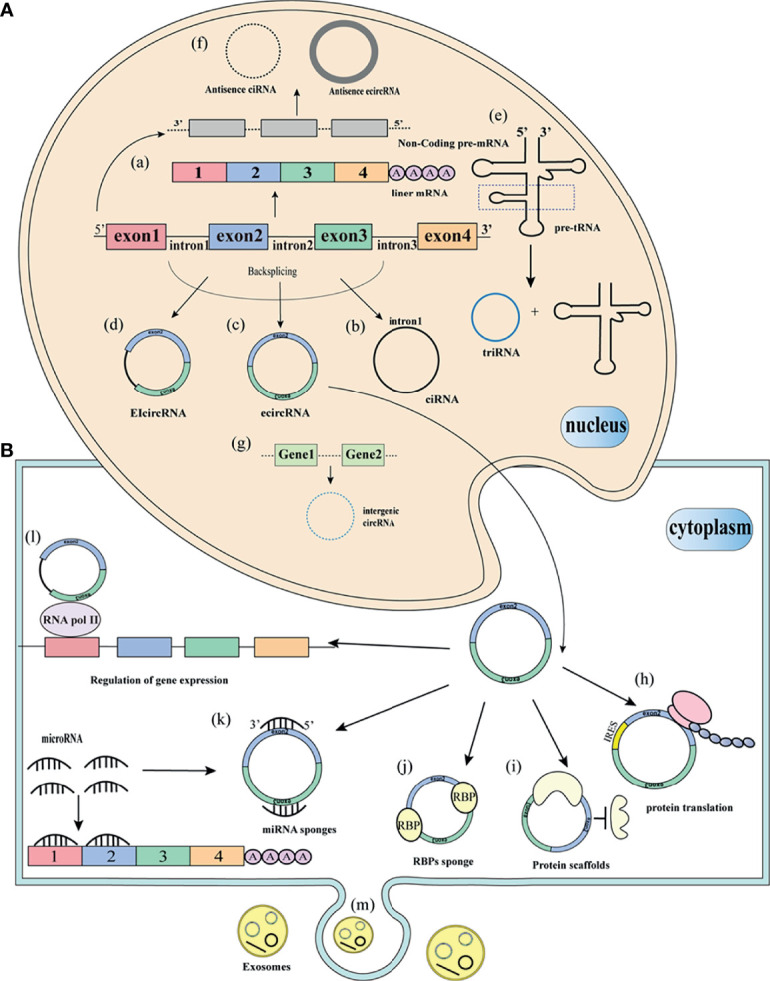
Biogenesis and function of circRNAs **(A)** Biogenesis of circRNAs. (a) Pre-mRNA splicing removes introns to form mature linear mRNA. (b) The introns removed by pre-mRNA splicing form circularization to form a stable ciRNA. (c) Circularization of pre-mRNA exons into ecircRNA. (d) pre-mRNA exons and introns are circularized into ecircRNAs. (e) removal of pre-tRNA introns, release to form triRNA and tRNA. (f) form antisense circRNAs from non-coding regions of pre-mRNA. (g) from two different intergenic sequences to form intergenic circRNAs. **(B)** Functions and of circRNAs. (h) circRNAs translation protein with similar IRES sequence. (i) circRNAs as protein scaffolds. (j) circRNAs bind to RBPs. (k) circRNAs as sponges for miRNA. (l) circRNAs interact with RNA polymerase II to regulate gene expression. (m) circRNAs are packaged into vesicles and released outside the cell to perform biological functions.

CircRNAs are derived from the reverse splicing mechanism of pre-mRNAs to form a single-stranded closed loop (16). However, the biogenesis mechanism has not been fully elucidated, circRNAs can be derived from exons, introns, 3’UTR, 5’UTR, intergenic regions, or antisense sequences (**Figure 1**). As early as 2013, Jeck et al. proposed two circRNAs cyclization models: Lariat-driven circularization and intron-pairing-driven circularization (13). Lariat-driven circularization, also known as exon skipping, is connected by non-adjacent exons as donors and acceptors to form a lasso structure. Then the introns in the lasso are removed, resulting in ecircRNAs. The biogenesis of circRNAs is mainly studied in ecircRNAs, other types of circRNAs are rarely studied. EcircRNAs account for 80% of identified circRNAs (17). The biogenesis of eicircRNAs is similar to ecircRNAs. In some cases, the intron portion is completely sheared to form eicircRNAs. Another model is intron-pairing-driven circularization, also known as direct reverse splicing. This model is based on the direct base pairing of the flanking introns, and then the introns are removed to form a ring structure (17). Zhang et al. reported a class of intron-derived circRNAs, namely CiRNAs (5). The formation of ciRNAs depends on the 7 nucleotides GU enrichment element near the 5’ splicing site and the 11 nucleotides C-rich element near the branch site. Zhang et al. believed that the exon cycle depends on the complementary sequences of the flanking introns. Intergenic circRNAs are formed from genes outside known genetic loci (11). TricRNA is formed by intron excision of the pre-tRNA by the tRNA splicing mechanism, followed by intron release and binding into tRNA and TricRNA (18).

In addition, multiple factors are involved in the biogenesis of circRNAs. Zhang et al. reported that the exon cycle depends on the complementary sequences of the flanking introns (19). Some RBPs play an important role in reverse splicings, such as MBL(splicing factor muscleblind), QKI(Quaking), and FUS(fused in sarcoma), binding to both sides of the flanking intron sequence enhances exon cycling by tightly linking the 3 ‘and 5’ ends of circRNAs. Thereby promoting exon circulation. Muscleblind is a splicing factor for MBL-derived genes and MBL in Drosophila promotes the production of circRNAs from the second exon of its own pre-mRNA by binding to flanking introns (20). The QKI of the STAR family is a tumor suppressor protein with three isoforms, all of which have the same KH domain but have different 3’UTRs. Among them, QKI-5, the most abundant nuclear isoform, acts on circRNAs during splicing. QKI dimerizes through its N-terminal Qua1 domain and binds to two-part sequence motifs that can be located on the same or separate RNA molecules (21). The investigation of PAR-CLIP cross-linking in human embryonic kidney cells (HEK293T) indicated that the majority of QKI binding occurs within introns and is responsible for circRNA synthesis, which limits proliferation and EMT during human cancer (22). In addition, Conn et al. also introduced consensus binding sequences for QKI in flanking introns to enable circRNAs to be generated from exons that normally only undergo canonical linear splicing (21). A recent study showed that overexpression of circ-SHPRH in cadmium-transformed BEAS-2B cells promoted the expression of QKI and significantly inhibited cell proliferation, EMT, invasion, migration, and non-anchored growth. Conclusions of Conn et al. (23). FUS was first reported to be involved in circRNA generation in the nervous system in 2017 (24). Cao et al. recently discovered that the nematode homologous gene FUST-1 promotes the creation of numerous circRNAs while having no effect on the analogous linear mRNA, regulating exon skipping and reverse splicing, surprisingly, CLIP-seq results suggest that FUS attaches to stem-loop secondary structure rather than particular sequences (25).

Besides, negative regulators destroy the stability of intron interactions, thereby reducing the cyclization efficiency, such as adenosine deaminases acting on RNA 1 (ADAR1) reduces the efficiency of cyclization by disrupting the base pairing between flanking introns through the A to I RNA editing mechanism (15). ADAR is an adenosine deaminase that is widely expressed in humans and can be applied to RNA modification. ADAR systems are used for programmable RNA editing *in vitro* and *in vivo* by recruiting ADARs to target RNA sequences using ADAR recruitment guide RNAs (adRNAs). Two recent studies have designed circRNAs that can recruit ADARs to improve RNA editing efficiency. Katrekar et al. engineered a highly stable circular ADAR-recruiting guide RNA (cadRNA) to recruit endogenous ADARs, improving the efficiency and durability of RNA editing (26). The engineered circ-arRNAs designed by Yi et al.’s LEAPER2.0 system have much higher editing efficiency than the corresponding linear arRNAs, which greatly improves the efficiency and robustness of RNA editing (27).

Interestingly, UAP56 and URH49 proteins can assist the transport of circRNAs from the nucleus to the cytoplasm (28). This discovery is novel, however, many of the regulatory factors involved in circRNAs biogenesis remain unclear and require more research. A better understanding of the biogenesis mechanism of circRNAs will lead to a better understanding of their specific roles in cancer development. Future studies can explore the levels of specific key factors that regulate the biogenesis of circRNAs, which will also provide innovative strategies for cancer treatment and prevention.

### 2.3 Biological Functions of CircRNAs

In addition to its unique way of formation, how circRNAs participate in the process of biogenesis has also deepened our understanding of circRNAs. However, the functions of most circRNAs are still unknown. Recent studies show that the functions of circRNAs are mainly ceRNA or miRNA sponging, binding with proteins, regulation of pre-RNA splicing, regulation of parental gene expression, and potential translation templates for proteins or peptides (**Figure 1**).

#### 2.3.1 Acting as CeRNA or MiRNA Sponging

MicroRNAs (miRNAs) are a class of small non-coding RNAs, which play a regulatory role in various cellular activities including cancer by pairing regulatory genes with mRNAs target bases. Hansen et al. first proposed the concept of miRNAs sponges in 2013 (29). There are miRNAs response elements on circRNAs, which can competitively bind to miRNAs, eliminate the inhibitory effect of miRNAs on target genes, and regulate the expression of related genes. They demonstrated for the first time that circCIRS-7 (CDR1as) can be a sponge of miRNAs. CIRS-7 promotes the progression of a variety of tumors. There are more than 70 miR-7 sponge binding sites on CIRS-7. CIRS-7 inhibits miR-7 and participates in various events in tumorigenesis, such as cell proliferation, migration, invasion, and differentiation (30). With the development of scientific research, more and more circRNAs have been found to act as sponges for miRNAs. This mechanism affects cell proliferation, migration, invasion, and blood vessel formation, and has been widely reported in the cancer field. For example, the combination of circSATB2 and miR-326 regulates the expression of FSCN1 and further promotes the proliferation, migration, and invasion of NSCLC cells (31). In addition, some circRNAs have also been found to combine multiple miRNAs to act on different systems. For example, cirMAT2B can be combined with miR-515-5p to increase the expression of HIF-1α and promote the growth of gastric cancer (32). Moreover, circMTO1 can also combine with miR-541-5p to inhibit the progression of liver cancer (33).

#### 2.3.2 Interaction With Proteins

Another function of circRNAs is to directly bind proteins to participate in physiological and pathological processes (**Figure 1**). More than 800 RNA-binding proteins (RBPs) have been identified in the human genome (34). RBPs play a role in circRNAs splicing, processing, folding, stabilization, and positioning. For example, CircFoxo3 is formed by Foxo3 exon 2 and has a wide range of complex biological functions, which are related to cell differentiation, apoptosis, and cycle. It has been reported that in the cytoplasm, the senescence-related proteins ID-1, E2F1, FAK, and HIF1α interact with circFoxo3 and no longer exert their anti-aging and anti-stress effects, leading to the promotion of cell senescence (35). CircFoxo3 can also bind to cyclin cells cyclin-dependent-kinase 2 (CDK2) and cyclin-dependent kinase inhibitor 1 (p21) to form a ternary complex to inhibit the binding of CDK2 and p21, and in the G1 phase inhibits the cell cycle progression (36). In addition, circMBL can bind to mannose-binding lectin (MBL) protein to control excess MBL protein (20). Two circRNAs, KIRKOS-73 and KIRKOS-71, are able to regulate the exosomal metastasis of p53 expression in recipient cells, and p53 plays a key role in metastasis and tumorigenesis (37). CircAgo2 transfers HuR protein from the nucleus to the cytoplasm, stabilizing the binding of mRNA and AU-rich elements in UTR (38). CircPABPN1 competitively binds to HuR, prevents HuR from binding to PABPN1 mRNA, and subsequently inhibits the translation of PABPN1 (39). The above studies have proved that the interaction between circRNAs and proteins plays an important role.

#### 2.3.3 Regulation of Pre-RNA Splicing

CircRNAs may affect the splicing of pre-RNA and can compete with pre-RNA for splicing sites. For example, circUBR5 may be involved in the RNA splicing regulation process, it can be combined with the splicing regulator QKI in the nucleus, NOVA alternative splicing regulator 1 (NOVA1), and U1 small nuclear RNA (snRNA) (40). CircSMARCA5 regulates VEGFA mRNA splicing and angiogenesis in glioblastoma multiforme through the binding of SRSF1 (41).

#### 2.3.4 Regulation of Gene Expression

EIciRNAs and ciRNAs are circRNAs with intron sequences, which are mainly located in the nucleus. Experiments have shown that EIciRNAs and ciRNAs can regulate gene expression. For example, the knockdown of circEIF3J and circPAIP2 can cause the transcription level of EIF3J and PAIP2 to decrease (42). EIciRNAs can promote the transcription of their parental genes in cis by interacting with U1 snRNA, revealing a new regulatory strategy for gene expression in RNA-RNA interactions (43). Li et al. found that ci-ankrd52 shows a different open structure conformation from pre-mRNA with the same sequence, which can replace pre-mRNA to form more stable R-loops (44). It can be seen that ci-ankrd52 plays a potential role in promoting transcription elongation. However, the effect of EIciRNAs and ciRNAs regulation still need to study in-depth.

#### 2.3.5 Translation Templates for Proteins or Peptides

Although circRNAs have long been considered non-coding RNAs that cannot translate proteins, recent studies have shown that circRNAs do not rely on conventional translation modes and have translation potential. Previous studies have found that some circRNAs have internal ribosome entry (IRE) site sequences or open reading frame (ORF) translatable proteins such as circMAPK1 and circMBL3. CircMAPK1 encodes a new protein with a length of 109 amino acids that competitively binds to MEK1 to inhibit the phosphorylation of MAPK1 (45). CircMBI translates to a small peptide in the head of a fly (46). However, recent studies have found that circRNAs lacking this sequence can also translate proteins. The N6-methyladenosine (M6A) modification allows circRNAs to be translated in a cap-independent manner (47). In addition, bioinformatics tools have been developed to predict translation potential, but the accuracy needs to be verified. Although these new discoveries are exciting, the function and efficiency of these translated proteins or peptides need further research (48, 49).

### 2.4 Identification of CircRNAs

Early RNA sequencing did not identify circular RNAs without a ployA tail, and non-linear fragments were often considered errors and were ignored. This section introduces some traditional circRNAs detection techniques and emerging technical methods (**Table 1**). Northern blot, qRT-PCR, RNA-seq, and Microarrays are examples of traditional circRNA detection techniques (63). However, previous RNA analysis methods are also difficult to study circRNAs. RNA-seq detection of RNA detection efficiency is low, so many low abundance circRNAs may be missed. And microarray technology has been used to detect linear RNA for a long time. The detection efficiency of circRNAs microarray is much higher than RNA-seq because it contains probes that target the head-to-tail connection (51). But it produces data that is difficult to compare between studies. Therefore, there is an urgent need to develop simple, effective, and sensitive new methods to study circRNAs.

**Table 1:** Detection of circRNAs.

Methods	Mechanism	Strengths	Weaknesses	Refs
Northern blot	Oligonucleotide probe capture	Distinguished between circRNAs and linear RNAs Estimated the circRNAs size	Low sensitivity and required large sample	(50)
Microarrays	Nucleic acid hybridization	High detection efficiency	Identified only known circRNAs	(51)
FISH	DNA probe hybridization	Subcellular localization of circRNAs	Expensive cost and time-consuming	(52)
RNA-seq	Transcript map	High sensitivity and specificity	Expensive equipment and reagents Complex operation process and data process	(53)
qRT-PCR	PCR amplification	High sensitivity and quantitative detection	Linear RNAs residue Amplified rolling loop error	(54)
ddqRT-PCR	PCR amplification based on Poisson distribution algorithm	High sensitivity and accuracy Simplified experimental process	Expensive equipment	(55)
Ligation-based PCR	PCR amplified DNA probe	Exonuclease and reverse transcription steps are not required Simple, high sensitivity, and specificity	Identified only one circRNA at a time	(56)
RT-RCA	Reverse transcriptase rolling cycle amplification	Simple operation, low cost, and high sensitivity	Complex process and time-consuming	(57)
LAMP	SLP induced doubleexponential amplification	High amplification efficiency, high sensitivity, and specificity Distinguished between circRNAs and linear RNAs	Complex process and expensive cost	(58)
circFL-seq	Rolling-cycle reverse transcription (RCRT) and nanopore sequencing	Identified and quantified full-length circular RNAs and isomer level Suitable for mass screening accurately	Identified fewer circRNAs isoforms	(59)
CIRI-long	RCRT	Resisted interference from residual linear RNAs	Less sensitive than full-length reads	(60)
isoCirc	RCA	Longer readings (up to 50 KB) High sensitivity	Expensive cost and false-positive	(61)
Electrochemical method	Back-splice junction (BSJ) and duplex-specific nuclease (DSN)	Avoided errors caused by additional RNase R process Super sensitivity and repeatability	Identified only specific circRNAs	(62)

Recently, a newly reported exome capture RNA sequencing technology can detect and characterize circRNAs in more than 2000 cancer samples (64). In addition, Zhang et al. developed a new method for quantitative detection of circular RNA with high sensitivity and specificity (56). The two cleverly designed DNA probes can be precisely connected by using ligase at the connection site of circular RNA. Distinguish circular RNA from corresponding linear RNA. Liu et al. proposed a reverse transcription-rolling cycle amplification (RT-RCA) process that can selectively amplify target circular RNA (57). Zhang et al. designed a pair of Stem-loop primers (SLPs) based on loop-mediated isothermal amplification (LAMP), an excellent nucleic acid amplification method, that could accurately recognize circRNA junction sequences, thereby establishing an SLP-induced dual amplification system (58).The exponential amplification method makes circRNA detection simple and accurate. Additionally, Li et al. designed an electrochemical method for the recognition and capture of circRNAs with hairpin probes, avoiding additional RNase R treatment, and the method exhibited good sensitivity and selectivity (62).

According to the characteristics of circRNAs, some detection methods for circRNAs have been developed, including traditional detection methods and newly developed methods, which have their own advantages and disadvantages. However, the way forward is clear, and the detection method of circRNAs still needs to be developed or improved, so that its sensitivity, specificity, rapidity, and convenience can be applied to biomedical research and clinical detection. All these make it possible for circRNAs to become diagnostic tools and therapeutic targets.

### 2.5 Online Database for CircRNAs Research

In recent years, with the intensive study of circRNAs, researchers have developed many high-quality online databases. This section introduces some databases that can be used for circRNAs research in **Table 2**. These artificially established databases are of great significance to the study of the biological functions of circRNAs. In addition to the online database listed in the table, in 2022, the University of Padova developed a circRNA function prediction software CRAFT, which can predict circRNA sequences and molecular interactions with miRNAs and RBPs, as well as their coding potential (81). Nevertheless, the existing circRNAs collection is largely limited to certain well-studied species, such as humans and mice. In addition, the current annotations and naming are rather incomplete. Most databases only use one or two resources for annotations. Searching for the same circRNAs has different naming methods in different databases, which increases the difficulty of studying circRNAs.

**Table 2:** Database for circRNAs research.

Database	URL	Specie	Function	Refs
CircBase	http://www.circbase.org/	Human, Mouse, Caenorhabditis elegan, Latimeria	Searched for circRNAs sequence	(14)
Circbank	http://www.circbank.cn/help.html	Human	Organized human circular RNA data in the CircBase database, and performed protein-coding potential and miRNA interaction prediction analysis based on sequence information	(48)
CircAtlas	http://circatlas.biols.ac.cn/	Human, Macaque, Mouse, Rat, Pig, Chicken	Fully annotated circRNAs and Assessed the relevance of circRNAs to various diseases	(49)
MiOncoCirc	https://mioncocirc.github.io/	Human	Associated circRNAs with cancer clinical symptoms and diseases	(64)
CircInteractome	http://circinteractome.nia.nih.gov/	Human, Fruitfly	Predicted the binding of circRNAs to RBP or miRNA and designed PCR primers and circRNAs specific siRNA	(65)
CircRNADb	http://reprod.njmu.edu.cn/circrnadb	Human	Predicted the binding of circRNAs to RBP or miRNA	(66)
StarBase	http://starbase.sysu.edu.cn/	Human, Mouse, Elegan	Analyzed miRNAs-circRNAs interactions to find potential microRNA targets	(67)
Circ2Traits	http://gyanxet-beta.com/circdb/	Human, Mouse, Elegan	Collected circRNAs related to human diseases and predicted the interaction between miRNA and human protein-coding genes, lncRNA and circRNAs	(68)
CircRNA disease	http://cgga.org.cn:9091/circRNADisease/	Human	Retrieved disease-related circRNAs information	(69)
CirclncRNAnet	http://app.cgu.edu.tw/circlnc/	Human	Annotated the multi-line function-related information of CircRNAs/LncRNAs	(70)
CSCD2	http://geneyun.net/CSCD2 or http://gb.whu.edu.cn/CSCD2	Human	An abundant circRNAs data volume, focusing on tumor-specific circRNAs expression and predicted potential full-length and open reading frame sequences of circRNAs	(71)
Deepbase	http://rna.sysu.edu.cn/deepBase/	Human, Mouse, Chicken, Pan troglodytes, Gorilla, Macaca mulatta, Bos Taurus	Annotated and identified circRNAs/miRNAs/piRNAs, etc. and their expression patterns	(72)
CIRCpedia	http://www.picb.ac.cn/rnomics/circpedia/	Human, Mouse, Rat, Fruitfly, Worm, zebrafish	Annotated circRNAs	(73)
TRCirc	http://www.licpathway.net/TRCirc/view/index	Human	Searched for the TFBS of circRNAs can help discover the transcriptional regulation mechanism of circRNAs	(74)
ExoRBase	http://www.exoRBase.org	Human	Retrieved circRNAs information expressed in peripheral blood exosomes	(75)
CircNet2.0	https://awi.cuhk.edu.cn/∼CircNet.	Human	Identified new circRNAs and integrated the circRNAs-miRNAs-mRNAs interaction network	(76)
CircR2Disease	http://bioinfo.snnu.edu.cn/CircR2Disease/	Human, Mouse, Rat	Searched for the relationship between circRNAs and disease in the literature	(77)
CirComPara2	https://github.com/egaffo/CirComPara2			(78)
circMine	http://hpcc.siat.ac.cn/circmine http://www.biomedical-web.com/circmine/	Human	Assessed the clinical and biological significance of circRNAs and predicted circRNA-miRNA interactions and circRNAs translatability	(79)
ViroidDB	https://viroids.org	viroids	Collected viroid-like circular RNA sequences	(80)

## 3 Roles of CircRNAs in Cancer

To date, a large number of reports have found that circRNAs are abnormally expressed in tumor tissues, and more and more evidence shows that circRNAs play a critical role in the occurrence and development of tumors (82, 83). Most of the abnormal circRNAs discovered are the sponges or proteins of miRNAs (**Table 2**). In addition to affecting cancer cell proliferation, migration, invasion, and escape from apoptosis and angiogenesis. CircRNAs can also regulate these cancer markers by regulating signal pathways such as Wnt/β-catenin (7), PIK3/AKT (84), and MAPK/ERK pathways (85). Among them, CIRS-7 is widely studied in cancer and is usually up-regulated in cancer cells. It has been described to be expressed in liver cancer, lung cancer, gastric cancer, colorectal cancer, breast cancer, and other cancers (30). The miRNA regulated by CIRS-7 are miR-7 (86), miR-135a-5p (87, 88), miR-1270 (89), miR-26a-5p (90) and so on.

**Figure 2:**
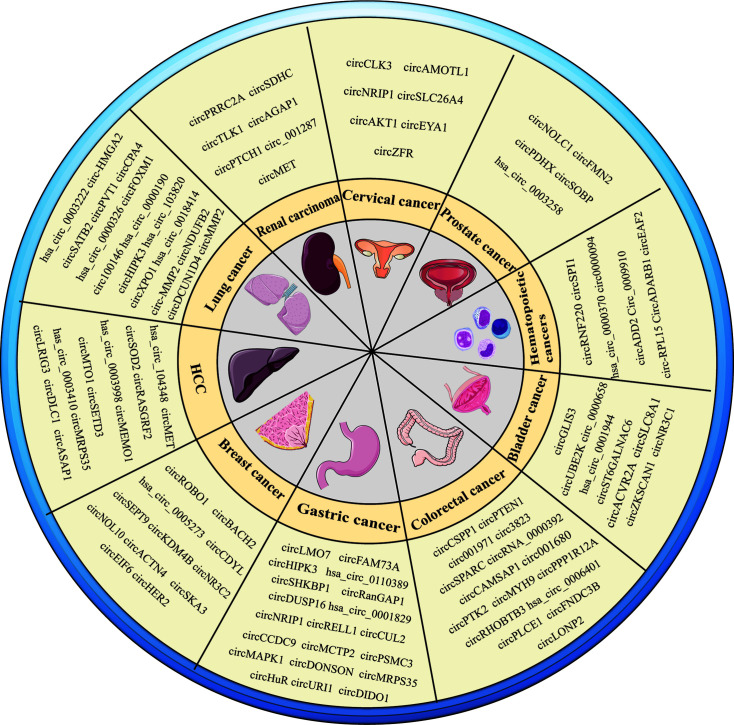
Overview of circRNAs in various types of cancers.

In addition to the circRNAs described above, some circRNAs expressed in common cancers have recently been discovered, as shown in **Table 3** and **Figure 2**.

**Table 3:** Dysregulated circRNAs in common cancer.

Cancer type	CircRNA	CircBase ID	Mechanism	Target	Expression in cancer	Function	Refs
**Lung cancer**	circ0003222	hsa_circ_0003222	MiRNA sponge	miR-527/PHF21B/β-catenin	up-regulated	Promote cell proliferation, invasion, and migration	(6)
	circHMGA2	hsa_circ_0027446	MiRNA sponge	miR-1236-3p/ZEB1	up-regulated	Promote cell metastasis and EMT	(91)
	circSATB2	hsa_circ_0118551	MiRNA sponge	miR-326/FSCN1	up-regulated	Promote cell proliferation, migration, and invasion	(31)
	circPVT1	hsa_circ_0085536	MiRNA sponge	miR-30d and miR-30e/cyclin F (CCNF)	up-regulated	Promotes LUSC progression	(92)
	circCPA4	hsa_circ_0082369	MiRNA sponge	miRNA let-7/PD-L1	up-regulated	Promote cell proliferation, mobility, and EMT	(93)
	circ0000326	hsa_circ_0000326	MiRNA sponge	miR-338-3/RAB14	up-regulated	Promote cell proliferation and migration Inhibit apoptosis	(94)
	circFOXM1		MiRNA sponge	miR-614/FAM83D	up-regulated	Promoted cell proliferation and cell cycle progression	(95)
	circ100146		MiRNA sponge	miR-361-3p and miR-615-5p/SF3B3	up-regulated	Promote cell proliferation and invasion Inhibit apoptosis	(96)
	circ0000190	hsa_circ_0000190	MiRNA sponge Regulation of gene expression	miR-142-5p/CDKs EGFR-MAPK-ERK	up-regulated	Promote cell proliferation, migration, and tumor growth	(97)
	circ103820	hsa_circ_0072309	MiRNA sponge	miR-200b-3p/LATS2 and SOCS6	down-regulated	Inhibit cell proliferation, migration, and invasion	(98)
	circ0018414	hsa_circ_0018414	MiRNA sponge	miR-6807-3p/DKK1	down-regulated	Inhibit cell proliferation Promote apoptosis	(99)
	circHIPK3	hsa_circ_0021592	MiRNA sponge	miR-124-3p-STAT3-PRKAA/AMPKa	down-regulated	Inhibit cell proliferation, migration, invasion, and autophagy	(100)
	circNDUFB2	hsa_circ_0082730	Protein scaffolds	RIM25/IGF2BPs	down-regulated	Inhibit cell proliferation and migration	(101)
	circDCUN1D4	hsa_circ_0126569	Protein scaffolds	HuR/TXNIP	down-regulated	Inhibit cell invasion and migration	(102)
	circXPO1	hsa_circ_0054899	Protein binding	IGF2BP1/CTNNB1	up-regulated	Promote tumor growth	(103)
	circMMP2	hsa_circ_0039411	Protein binding	IGF2BP3/FOXM1	up-regulated	Promote cell proliferation, migration, and EMT	(104)
**Colorectal cancer**	circCSPP1	hsa_circ_0001806	MiRNA sponge	miR-431/ROCK1/ZEB1	up-regulated	Promote cell proliferation, migration, and invasion	(105)
	circ001971		MiRNA sponge	miR-29c-3p	up-regulated	Promote cell proliferation, invasion, and angiogenesis	(106)
	circ3823		MiRNA sponge	miR-30c-5p/TCF7	up-regulated	Promote cell proliferation, metastasis, and angiogenesis	(107)
	circSPARC		MiRNA sponge	miR-485-3p/JAK2/STAT3	up-regulated	Promote cell migration and proliferation	(108)
	circCAMSAP1	hsa_circ_0001900	MiRNA sponge	miR-328-5p/E2F1	up-regulated	Promote tumor growth	(109)
	circ001680		MiRNA sponge	miR-340/BMI1	up-regulated	Promote cell proliferation and migration	(110)
	circCUL2		MiRNA sponge	miR-208a-3p/PPP6C	down-regulated	Inhibit cell proliferation Promote apoptosis and autophagy	(9)
	circPTEN1		Protein binding	Smad4/TGF-β/Smad	down-regulated	Inhibit cell metastasis and invasion	(111)
	circPTK2	hsa_circ_0005273	Protein binding	vimentin	up-regulated	Promote cell proliferation, metastasis, and EMT	(112)
	circMYH9		Protein scaffolds	hnRNPA2B1/p53	up-regulated	Promote cell proliferation	(113)
	circPPP1R12A	hsa_circ_0000423	Protein code	circPPP1R12A-73aa/hippoyap	up-regulated	Promote cell proliferation, migration, and invasion	(114)
	circRHOBTB3	hsa_circ_0073431	Protein binding	HuR/PTBP1	down-regulated	Inhibit cell metastasis, and invasion	(115)
	circ0006401	hsa_circ_0006401	Protein code	col6a3	up-regulated	Promote cell proliferation and migration	(116)
	circPLCE1		Protein code	circPLCE1-411/HSP90α/RPS3/NF-κB	down-regulated	Inhibit cell proliferation and metastasis	(117)
	circFNDC3B		Protein code	circFNDC3B-218aa	down-regulated	Inhibit cell proliferation, migration, and invasion	(118)
	circLONP2	hsa_circ_0008558	Transcriptional regulation	DGCR8/Drosha/miR-17	up-regulated	Promote cell invasion and metastasis	(119)
**Hepatocellular carcinoma**	circ104348		MiRNA sponge	miR-187-3p/RTKN2/Wnt/β-catenin	up-regulated	Promote cell proliferation, migration, and invasion Inhibit cell apoptosis	(7)
	circASAP1	hsa_circ_0085616	MiRNA sponge	miR-326/miR-532-5p-MAPK1/CSF-1	up-regulated	Promote cell proliferation, colony formation, migration, and invasion	(120)
	circMET	hsa_circ_0082002	MiRNA sponge	miR-30-5p/Snail/DPP4/CXCL10	up-regulated	Promote cell invasion and EMT	(121)
	circSOD2		MiRNA sponge	miR-502-5p/DNMT3a/JAK2/STAT3/	up-regulated	Promote cell proliferation and invasion	(122)
	circRASGRF2	hsa_circ_0073181	MiRNA sponge	miR-1224/FAK	up-regulated	Promote cell proliferation and migration	(123)
	circ0003998	hsa_circ_0003998	MiRNA sponge	miR-143-3p/FOSL2	up-regulated	Promote invasion	(124)
	circMEMO1		MiRNA sponge	miR-106b-5p/TCF21	down-regulated	Inhibit invasion and metastasis	(125)
	circMTO1		MiRNA sponge	miR-541-5p/ZIC1/Wnt/β-catenin	down-regulated	Inhibit cell proliferation, migration, and invasion	(33)
	circSETD3	hsa_circRNA_0000567 /hsa_circRNA_101436	MiRNA sponge	miR-421/MAPK14	down-regulated	Inhibit cell proliferation	(126)
	circ0003410	hsa_circ_0003410	MiRNA sponge	miR-1393p/CCL5	up-regulated	Promote cell proliferation and migration	(127)
	circMRPS35	hsa_circ_0000384	MiRNA sponge Protein code	miR-148a -3p/STX3/PTEN circMRPS35-168aa	up-regulated	Promote cell proliferation, migration, invasion, clone formation, and cell cycle	(128)
	circLRIG3	hsa_circ_0027345	Protein scaffolds	EZH2/STAT3	up-regulated	Promote cell proliferation, migration, and invasion Inhibit apoptosis	(129)
	circDLC1		Protein binding	HuR/MMP1	down-regulated	Inhibit cell proliferation and motility	(130)
**Gastric cancer**	circLMO7	hsa_circ_0008259	MiRNA sponge	miR-30a-3p/WNT2/β-Catenin	up-regulated	Promote cell proliferation, migration, invasion, and metastasis	(131)
	circFAM73A		MiRNA sponge	miR-490-3p/HMGA2	up-regulated	Promote cell proliferation, migration	(132)
	circHIPK3	hsa_circ_0021592	MiRNA sponge	miR-637/AKT1	up-regulated	Promote cell proliferation	(8)
	circ0110389	hsa_circ_0110389	MiRNA sponge	miR-127-5p/miR-136-5p-SORT1	up-regulated	Promote cell proliferation, migration, and invasion	(133)
	circSHKBP1	hsa_circ_0000936	MiRNA sponge	miR-582-3p/HUR/VEGF	up-regulated	Promote cell proliferation, Migration, invasion, and angiogenesis.	(134)
	circRanGAP1	hsa_circ_0063535	MiRNA sponge	miR-877-3p/VEGFA	up-regulated	Promote cell invasion and metastasis	(135)
	circDUSP16	hsa_circ_0003855	MiRNA sponge	miR-145-5p/IVNS1ABP	up-regulated	Promote tumorigenesis and invasion	(136)
	circ0001829	hsa_circ_0001829	MiRNA sponge	miR-155-5p-SMAD2	up-regulated	Promote cell proliferation, migration, and invasion	(137)
	circNRIP1	hsa_circ_0061275	MiRNA sponge	miR-149-5p/AKT1/mTOR	up-regulated	Promote cell autophagy, migration, invasion, and EMT	(138)
	circRELL1	hsa_circ_0001400	MiRNA sponge	miR-637/EPHB3	down-regulated	Inhibit cell proliferation, migration, invasion, and apoptosis	(139)
	circCUL2	hsa_circ_0018193	MiRNA sponge	miR-142-3p/ROCK2	down-regulated	Promote cell autophagy Inhibit cell proliferation, migration, and invasion	(140)
	circCCDC9	hsa_circ_0051667	MiRNA sponge	miR-6792-3p/CAV1	down-regulated	Inhibit cell proliferation, migration. and invasion	(141)
	circMCTP2	hsa_circ_0000657	MiRNA sponge	miR-99a-5p/MTMR3	down-regulated	Inhibit cell proliferation, migration, invasion, and metastasis	(142)
	circPSMC3	hsa_circ_0021989	MiRNA sponge	miR-296-5p/PTEN	down-regulated	Inhibit cell proliferation and metastasis	(143)
	circMAPK1	hsa_circ_0004872	MiRNA sponge	miR-224/Smad4/ADAR1	down-regulated	Inhibit cell proliferation, migration, and invasion	(45)
	circDONSON	hsa_circ_0061550	Protein code	MAPK1-109aa/MEK1/MAPK1	down-regulated	Inhibit cell proliferation, migration	(144)
			Interaction with proteins	SNF2L/SOX4	up-regulated	Promote cell proliferation, migration, invasion, and metastasis	(145)
	circMRPS35	hsa_circ_0025733	Protein modification	KAT7/FOXO1/3a	down-regulated	Inhibit cell proliferation, invasion	(146)
	circHuR	hsa_circ_0049027	Protein binding	CNBP/HuR	down-regulated	Inhibit cell growth, invasion, and metastasis	(147)
	circURI1	hsa_circ_0050333	Transcriptional regulation	hnRNPM	down-regulated	Inhibit cell migration, invasion, and metastasis	(148)
	circDIDO1	hsa_circ_0061137	Protein code and Interaction with proteins	529aa/PARP1,PRDX2	down-regulated	Inhibit cell proliferation, migration, and invasion	(149)
**Breast cancer**	circROBO1		MiRNA sponge	miR-217-5p/KLF5/FUS	up-regulated	Promote cell proliferation, migration, and invasion	(150)
	circBACH2		MiRNA sponge	miR-186-5p/miR-548c-3p/CXCR4	up-regulated	Promote cell proliferation, migration, and invasion	(151)
	circ0005273	hsa_circ_0005273	MiRNA sponge	miR-200a-3p//YAP1	up-regulated	Promote cell proliferation and migration	(152)
	circCDYL		MiRNA sponge	miR-1275-ATG7/ULK1	up-regulated	Promote autophagy and malignant progression	(153)
	circSEPT9		MiRNA sponge	miR-637/LIF/Stat3	up-regulated	Promote cell proliferation, migration, and invasion Inhibit apoptosis	(154)
	circKDM4B	hsa_circ_0002926	MiRNA sponge	miR-675/NEDD4L/PI3KCA/PI3K/AKT and VEGFA	down-regulated	Inhibit cell migration and invasion	(155)
	circNR3C2	hsa_circ_0071127	MiRNA sponge	miR-513a-3p/HRD1/Vimentin	down-regulated	Inhibit cell proliferation migration, invasion, and EMT	(156)
	circNOL10		MiRNA sponge	miR-767-5p/SOCS2/JAK2/STAT5	down-regulated	Inhibit cell proliferation, migration, invasion, and EMT	(157)
	circACTN4		Protein binding	FUBP1/MYC	up-regulated	promote cell growth, invasion, and metastasis	(158)
	circSKA3		Protein binding	Tks5/integrin β1	up-regulated	Promote cell invasion	(159)
	circEIF6		Peptide code	EIF6-224aa/MYH9/Wnt/β -catenin	up-regulated	promote cell proliferation and migration	(160)
	circHER2		Protein code	HER2-103/EGFR	up-regulated	Promote cells proliferation, invasion, and tumorigenesis	(161)
**Hematopoietic cancers**	circRNF220	hsa_circ_0012152	MiRNA sponge	miR-30a/MYSM1/IER2	up-regulated	Promote cell proliferation Inhibit cell apoptosis	(162)
	circSPI1		MiRNA sponge Interaction with proteins	miR-1307-3p、miR-382-5p and miR-767-5p eIF4AIII	up-regulated	Promote cell proliferation and inhibit cell apoptosis	(163)
	circ0000370	hsa_circ_0000370	MiRNA sponge	miR-1299/S100A7A	up-regulated	Increase cell viability and inhibit apoptosis	(164)
	circ0000094	hsa_circ_0000094	MiRNA sponge	miR-223-3p/FBW7	down-regulated	Inhibit cell proliferation、migration and invasion Promote cell apoptosis	(165)
	circADD2		MiRNA sponge	miR-149-5p/AKT2	down-regulated	Inhibit cell proliferation and promote cell apoptosis	(166)
	circ0009910		MiRNA sponge	miR-34a-5p/ULK1	up-regulated	Promote cell autophagy	(167)
	circRPL15	hsa_circ_0064574	MiRNA sponge	miR146b-3p/RAF1	up-regulated	Promote cell viability	(168)
	circADARB1		MiRNA sponge	miR-214-3p/p-Stat3	up-regulated	Promote cell proliferation Inhibit cell apoptosis	(169)
	circEAF2		MiRNA sponge	miR-BART19-3p/APC/β-catenin	down-regulated	Promote cell apoptosis and inhibit tumor progression	(170)
**Renal carcinoma**	circPRRC2A		MiRNA sponge	miR-514a-5p and miR-6776-5p/TRPM3	up-regulated	Promote EMT and invasion	(171)
	circSDHC	hsa_circ_0015004	MiRNA sponge	miR-127-3p/CDKN3/E2F1	up-regulated	Promote cells proliferation, and invasion	(172)
	circTLK1		MiRNA sponge	miR-136-5p/CBX4	up-regulated	Promote cell proliferation, migration, and invasion	(173)
	circAGAP1		MiRNA sponge	miR-15-5p/E2F	up-regulated	Promote cell proliferation, migration, and invasion	(174)
	circPTCH1		MiRNA sponge	miR-485-5p/MMP14	up-regulated	Promote RCC metastasis and EMT	(175)
	circ001287		MiRNA sponge	miR-144/CEP55	up-regulated	Promote cell proliferation, migration, and invasion	(176)
	circMET	hsa_circ_0082002	MiRNA sponge	miR1197/SMAD3	up-regulated	Promote cell proliferation, and tumor progression	(177)
			Protein binding	YTHDF2/CDKN2A			
**Bladder Cancer**	circGLIS3	hsa_circ_0002874	MiRNA sponge	miR-1273f/SKP1/Cyclin D1	up-regulated	Promote cell proliferation, migration, and invasion	(178)
	circUBE2K	hsa_circ_0009154	MiRNA sponge	miR-516b-5p/ARHGAP5/RhoA	up-regulated	Promote cell proliferation, migration, and invasion	(179)
	circ0000658		MiRNA sponge	miR-498/HMGA2	up-regulated	Promote cell proliferation, migration, invasion, and EMT	(180)
	circ0001944	hsa_circ_0001944	MiRNA sponge	miR-548/PROK2	up-regulated	Promote cell invasion and proliferation	(181)
	circST6GALNAC6		MiRNA sponge	STMN1/STMN1/EMT	down-regulated	Inhibit cell proliferation, migration, invasion, and EMT	(182)
	circACVR2A	hsa_circ_0001073	MiRNA sponge	miR-626/EYA4	down-regulated	Inhibit cell proliferation and metastasis	(183)
	circSLC8A1		MiRNA sponge	miR-130b, miR-494/PTEN	down-regulated	Inhibit cell proliferation, migration, and invasion	(184)
	circZKSCAN1		MiRNA sponge	miR-1178-3p/p21	down-regulated	Inhibit cell proliferation, migration, invasion, and metastasis	(185)
	circNR3C1		Protein binding	BRD4/C-myc/EZH2	down-regulated	Inhibit BC progression	(186)
	circNOLC1		MiRNA sponge	miR-647/PAQR4	up-regulated	Promote cell proliferation and migration	(187)
**Prostate cancer**	circFMN2		MiRNA sponge	miR-1238/LHX2	up-regulated	Promote cell proliferation, migration, and invasion	(188)
	circPDHX	hsa_circ_0003768	MiRNA sponge	miR-378a-3p/IGF1R	up-regulated	Promote cell proliferation and invasion	(189)
	circSOBP		MiRNA sponge	miR-141-3p/MYPT1/p-MLC2	down-regulated	Inhibit cell migration and invasion	(190)
	circ0003258	hsa_circ_0003258	MiRNA sponge Interaction with proteins	miR-653-5p/ARHGAP5 IGF2BP3/HDAC4	up-regulated	Promote cell migration and EMT	(191)
**Cervical cancer**	circCLK3		MiRNA sponge	miR-320a/FoxM1	up-regulated	Promote cell growth, migration, invasion, and metastasis	(192)
	circAMOTL1	hsa_circ_0004214	MiRNA sponge	miR-485-5p/AMOTL1	up-regulated	Promote cell proliferation and migration	(193)
	circNRIP1	hsa_circ_0004771	MiRNA sponge	miR-629-3p/PTP4A1/ERK1/2	up-regulated	Promote cell proliferation, migration, and invasion	(194)
	circSLC26A4		MiRNA sponge	miR-1287-5p/HOXA7	up-regulated	Promote cell proliferation, migration, and invasion	(195)
	circAKT1		MiRNA sponge	miR-942-5p/AKT1	up-regulated	Promote cell proliferation, and invasion	(196)
	circEYA1		MiRNA sponge	miR-582-3p/CXCL14	down-regulated	Promote cell apoptosis	(197)
	circZFR	hsa_circ_0072088	Interaction with proteins	SSBP1/CDK2/cyclin E1	up-regulated	Promote cell proliferation, invasion, and tumor growth	(198)

### 3.1 Lung Cancer

Lung cancer is the malignant tumor with the highest mortality rate in the world (199). Circ0003222 sponges miR-527 to down-regulate the expression of PHF21B and its downstream β-catenin. Thereby promoting the proliferation, migration, and invasion of tumor cells. Yu et al. (91) discovered a new type of circHMGA2 (hsa_circ_0027446) molecule through microarray, which is highly expressed in lung adenocarcinoma (LUAD). Mechanically, circHMGA2 promotes LUAD cell metastasis through the miR-1236-3p/ZEB1 axis. Yao et al. (99) found that circ_0018414 was down-regulated in LUAD tissues and cells, and inhibited the progression of LUAD through the Wnt/β-catenin pathway of miR-6807-3p/DKK1 axis inactivation. Some circRNAs have been described as binding to proteins in lung cancer. For example, circNDUFB2 (101), which is down-regulated in non-small cell lung cancer, acts as a scaffold to enhance the interaction between TRIM25 and IGF2BPs. It inhibits the growth and metastasis of NSCLC cells by regulating protein ubiquitination and degradation and cellular immune responses. In addition, Huang et al. (103)found that circXPO1 is highly expressed in LUAD through RNA sequencing. In terms of mechanism, circXPO1 can bind to IGF2BP1 to enhance the stability of CTNNB1 mRNA, thereby promoting the progress of LUAD.

The development of new NSCLC-specific biomarkers to aid in diagnosis and clinical decision-making has always been a pressing concern. Li et al. found that circ0003222 is related to the staging, metastasis, and survival rate of patients with non-small cell lung cancer (NSCLC) (6). Additionally, high expression of circ 0070354 was demonstrated to be substantially linked to advanced TNM staging and poor differentiation in NSCLC and was an independent predictor of poor prognosis. CEA, SCC, and Cyfra21-1 are the acronyms for CEA, SCC, and Cyfra21-1, respectively. The AUC of circ0070354, when combined with the other three mature tumor markers, was 0.730, which was much higher than the solitary diagnosis (200). According to the findings, some circRNAs potentially outperform traditional tumor markers in terms of diagnosis, and the combined diagnosis has higher sensitivity and specificity for lung cancer diagnosis and treatment.

### 3.2 Colorectal Cancer

Colorectal cancer (CRC) is the second leading cause of death from cancer worldwide (199). Jian et al. (110) tested the gene expression in 42 pairs of colorectal cancer tissues and normal tissues adjacent to cancer. The results showed that circ001680 was overexpressed in 71.4% of colorectal cancer tissues. In terms of mechanism, circ001680 promotes the proliferation and migration of colorectal cancer cells by targeting miR-340. Yang et al. discovered a new circRNA, circPTK2, and found that circPTK2 binds to the Ser38, Ser55, and Ser82 sites of vimentin protein to promote EMT of CRC cells *in vivo* and *in vitro (* 112). In addition to interacting with proteins to regulate the expression of target genes, circRNAs encoding proteins or peptides have also been found in colorectal cancer, such as circ0006401 (116), circPLCE1 (117), and circFNDC3B (118). Among them, circ000641 encoding peptide fragment promotes the proliferation and migration of CRC and promotes the stability of the host gene col6a3 mRNA, and thus promotes the proliferation and metastasis of CRC. The circPLCE1-411 protein encoded by circPLCE1 combined with the HSP90α/RPS3 complex plays a key role in the NF-κB activation of CRC and ultimately inhibits tumor proliferation and metastasis in CRC cells (117). The tumor suppressor circFNDC3B is mainly located in the cytoplasm and encodes a new protein circFNDC3B-218aa, thereby inhibiting the proliferation, invasion, and migration of colon cancer cells (118).

Wang et al. (108) found that circSPARC is highly expressed in the tissues and plasma of CRC patients, is associated with advanced TNM staging, lymph node metastasis, and a low survival rate of CRC. Mechanistically, circSPARC can upregulate the expression of JAK2 by sponge miR-485-3p, and ultimately promote the accumulation of phosphorylated p-STAT3, thereby promoting the proliferation and migration of cancer cells. The most commonly used colorectal tumor marker CEA has limited sensitivity in early CRC (201, 202). While circRNAs can be employed as reliable biomarker complements for CEA in CRC early diagnosis and treatment monitoring. According to the ROC curve analysis of Hu et al., the AUC (0.831 vs 0.657), sensitivity (0.677 vs 0.532), and specificity (0.915 vs 0.675) values of circ 001659 in the early diagnosis of CRC were higher than those of CEA as a novel biomarker of successful treatment and response for cancer tracking thing (203). These findings indicate that circRNAs can become potential diagnostic and prognostic biomarkers and therapeutic targets for the treatment of CRC.

### 3.3 Hepatocellular Carcinoma

Hepatocellular carcinoma (HCC) is the third leading cause of cancer-related deaths. There have been multiple reports that a variety of circRNAs inhibit or promote tumor progression in liver cancer. Hu et al. (120) found that circASAP1 promotes the proliferation and invasion of liver cancer cells by regulating the miR-326/miR-532-5p-MAPK1 signaling pathway, and then mediates tumor-associated macrophages by regulating the miR-326/miR-532-5p-CSF-1 pathway Cell infiltration. The circRNAs array analyzes the expression of circRNAs in tumor tissues and normal tissues. In a study by Dong et al. (125), it was found that 28 up-regulated and 18 down-regulated circRNAs were found in liver cancer tissues. circMEMO1 is significantly down-regulated in HCC samples and can act as a sponge of miR-106b-5p to regulate TCF21 promoter methylation and gene expression, thereby regulating HCC progression. Li et al. (128)found that circMRPS35 was highly expressed in 35 pairs of HCC patients compared with normal tissues. It is worth noting that circMRPS35 can not only adsorb miR-148a-3p, regulate the expression of Syntaxin 3 (STX3), thereby regulating the ubiquitination and degradation of phosphatase and tensin homolog (PTEN) but can also encode a peptide (circMRPS35-168aa), this peptide promotes cisplatin resistance in HCC cells. CircLRIG3 is significantly up-regulated in HCC, forming a ternary complex with EZH2 and STAT3, promoting EZH2-induced STAT3 methylation and subsequent phosphorylation, leading to the activation of STAT3 signal, thereby promoting the proliferation, migration, and invasion of liver cancer cells. Reduce cell apoptosis (129).

Wei et al. reported that the expression of circCDYL or the combined expression of HDGF and HIF1AN are independent markers for distinguishing early HCC, providing the possibility for the detection and early treatment of liver cancer (204). Yang et al. found that circFN1 promotes sorafenib resistance by regulating the miR-1205/E2F1 signaling pathway, which is a potentially valuable target for HCC resistance (205).

### 3.4 Gastric Cancer

Gastric cancer (GC) is the fourth leading cause of death in the world (199), especially in Asian countries, the incidence of gastric cancer is increasing year by year. Cao et al. found that circ0008259 (circLMO7) is highly expressed in GC tissues, circLMO7 sponge miR-30a-3p regulates the WNT2/β-Catenin pathway and affects the glutamine metabolism of GC cells, and ultimately promotes the growth and migration of GC (131). Peng et al. found that the level of circCUL2 in GC tissues and cells was significantly reduced, and the sponge engulfed miR-142-3p to regulate ROCK2, thereby inhibiting malignant transformation and inhibiting tumorigenicity *in vivo (* 140). In addition, Yan et al. (206) found that circEVI5 was significantly down-regulated in GC tissues and cells. circEVI5 sponges swallowed miR-4793-3p and increased the expression level of FOXO1 to inhibit the proliferation of GC and delay the cell cycle. Wang et al. (148)analyzed the circRNAs of five pairs of human stomachs and corresponding non-tumor adjacent specimens and found that circURI1 was significantly highly expressed in GC and metastasized in GC. It regulates a small part of genes involved in cell movement by isolating hnRNPM protein to inhibit GC metastasis. Zhang et al. (149) found that circDIDO1 is down-regulated in gastric cancer tissues, and its low level is associated with larger tumors, distant metastasis, and poor prognosis. In mechanism, circDIDO1 encodes a new 529aa protein, which interacts with poly ADP-ribose polymerase 1 (PARP1). Effect and inhibit its activity. Interestingly, circDIDO1 also binds to peroxide reduction protein 2 (PRDX2), which promotes the ubiquitination and degradation of PRDX2 mediated by rbx1, leading to inactivation of its downstream signaling pathways.

Further, numerous research has explored the clinical utility of circRNAs as biomarkers for the early detection and prognosis of gastric cancer. For instance, Song et al. (207) detected the expression profile of circRNAs and found that hsa_circ_000780 was significantly downregulated in GC tissue samples, and its level was correlated with the level of tumor clinicopathological features. Interestingly, circ000780 was also found in gastric juice of patients with early GC. In another report, circERBB2 (208) in plasma can be used as a prognostic biomarker for gastric cancer patients. CircERBB2 levels in preoperative plasma (high group) were significantly correlated with lymph node metastasis (P = 0.035), suggesting that it could be used to predict noninvasively the prognosis of GC.

### 3.5 Breast Cancer

Breast cancer (BC) is the main cause of cancer in women and the main cause of death in women. Wang et al. (151) used the circRNAs microarray data set and found that four circRNAs were abnormally expressed in TNBC. Among them, circBACH2 is most significantly elevated in BC tissues, and its high expression promotes epithelial-mesenchymal transition and cell proliferation and is positively correlated with the malignant progression of BC patients. Mechanistically, circBACH2 sponges miR-186-5p and miR-548c-3p, thereby releasing the expression of C-X-C chemokine receptor type 4 (CXCR4). Li et al. (160) discovered that circ-EIF6 encodes a new peptide called EIF6-224 amino acid (aa). EIF6-224aa directly interacts with the oncogene MYH9 in BC and inhibits the ubiquitin-proteasome pathway and subsequently activates the Wnt/β-catenin pathway to reduce the degradation of MYH9, thereby playing a carcinogenic effect. In addition, Wang et al. reported that circACTN4 can competitively bind to far upstream element-binding protein 1 (FUBP1) to prevent FUBP1 from binding to FIR, thereby activating MYC transcription and promoting tumor progression in breast cancer (158).

Some potential circRNAs biomarkers for early diagnosis of BC and prediction of recurrence and metastasis have emerged from the detection of clinical samples in the tissues and peripheral blood of BC patients and healthy controls, combined with the correlation analysis of clinicopathological factors and the analysis of prognosis and survival. For example, CircSMARCA5 can form an R-loop with its parental locus, causing a transcriptional pause at SMARCA5 exon 15, and SMARCA5 DNA is involved in chromatin remodeling in damaged regions.circSMARCA5 may serve as a therapeutic target for patients with drug-resistant BC (209). The above studies have provided new insights into the role of circRNAs in BC.

### 3.6 Hematopoietic Cancers

According to recent research findings, the involvement of circRNAs in hematological malignancies is becoming more widely recognized (210). Among them, aberrant circRNAs expression might upset the balance between self-renewal and differentiation of hematopoietic stem cells (HSCs), resulting in bone marrow failure or hematological malignancies.

Acute myeloid leukemia (AML), the most common leukemia in adults, is uncommon in children, but has a poor prognosis and is prone to relapse (211). Liu et al. discovered that circRNF220 is abundantly and precisely expressed in children’s peripheral blood and bone marrow using microarray technology. CircRNF220 knockout can reduce the proliferation of AML cell lines and primary cells while also promoting cell death (162). FLT3-ITD+ AML is a significant subtype of AML, and Zhang et al. observed that the effect of circ0000370 on the development of FLT3-ITD-positive AML may be directly connected to miR-1299 and S100A7A (164). Acute lymphoblastic leukemia (ALL) is the most prevalent malignant tumor in children, and circ0000094 has been demonstrated to be a molecular sponge of miR-223-3p, which can upregulate the expression of FBW7 by limiting the expression of miR-223-3p, hence preventing ALL progression (165). Zhu et al. found that circADD2, as a tumor suppressor gene in ALL, inhibited cell proliferation and promoted cell apoptosis both *in vitro* and *in vivo*. Mechanistically, circADD2, which can sponge miR-149-5p, may serve as a potential biomarker for ALL diagnosis or treatment (166). Interestingly, circRNAs expression profiles can also clearly distinguish Acute leukemia (AL). For example, Guo et al. reported circ0001857 and circ0012152 ALL and AML (212). The recently discovered Circ0009910 can regulate ULK1-induces autophagy by targeting miR-34a-5p and accelerating the resistance of CML cells to imatinib (167). High expression of circ-RPL15 was positively correlated with IGHV mutation status, which is crucial for evaluating CLL prognosis. MiR-146b-3p-mediated RAS/RAF1/MEK/ERK pathway inhibition could be alleviated by circRPL15 overexpression in CLL. CircRPL15 may represent a promising novel plasma biomarker for the diagnosis of CLL (168). Mei et al. found that the relative expression of circADARB1 was significantly increased in the plasma of Natural killer/T-cell lymphoma (NKTCL), which binds to miR-214-3p and regulates p-Stat3, promotes the proliferation of NKTCL cells, and inhibits apoptosis (169). Furthermore, Zhao et al. reported that CircEAF2 inhibited Epstein-Barr infection positive diffuse large B cell expansion and advanced apoptosis *via* the miR-BART19-3p/APC/-catenin axis (170).

Some of the circRNAs reported above play a regulatory role in the occurrence and development of hematological tumors through various molecular mechanisms, suggesting some potentials of circRNAs in the research of hematological malignancies in the future. These findings also aid in the diagnosis and prediction of hematological malignancies.

### 3.7 Other Types of Cancer

The abnormal expression of many circRNAs has been verified in many cancers. In renal cell carcinoma (RCC), Cen et al. found that circSDHC competitively binds to miR-127-3p, preventing it from inhibiting the downstream genes CDKN3 and E2F1 pathways, leading to RCC Malignant progress (172). NONO-TFE3 TRCC (Xp11.2 translocation/NONO-TFE3 fusion renal cell carcinoma) is a subgroup of renal cell carcinoma. Yang et al. found that highly expressed circMET accelerates the decay of CDKN2A mRNA by recruiting YTHDF2, while competitively binding miR- 1197, Regulates SMAD3 expression (177). In bladder cancer (BC), Yang et al. (179) used high-throughput sequencing and RT-qPCR to verify the abnormally high expression of circUBE2K BC tissue. As a ceRNA, the expression of ARHGAP5 was regulated by sponge miR-516b-5p to promote tumor development. The down-regulated circZKSCAN1 in BC tissues and cell lines up-regulates the expression of p21 through sponge miR-1178-3p, which inhibits the proliferation, migration, and invasion of bladder cancer (185). In addition, some circRNAs have also been found to play an important role in prostate cancer and cervical cancer, as shown in **Table 3**.

In short, various studies have shown that circRNAs are involved in the occurrence and development of various cancers. However, the role of circRNAs in the diagnosis and treatment of cancers needs to be further studied.

## 4 CircRNAs as Liquid Biopsy Biomarkers

There are RNase in human body fluids, and circRNAs can resist this enzyme, thus being a stable biomarker for the detection of body fluids such as blood, exosomes, saliva, and urine (213). In addition, the half-life of circRNAs in the blood is longer than that of mRNA. Coupled with the high abundance and specificity of circRNAs, circRNAs are expected to become an excellent non-invasive biomarker for tumor diagnosis and prognosis. Xu et al. reported that compared with breast cancer and adjacent normal tissues, the expression of circRNAs in peripheral blood was significantly higher than that of host genes (209). This discovery helps to explore diagnostic biomarkers for breast cancer.

CircRNAs may be used as biomarkers for cancer diagnosis and prognosis. It is worth noting that exosomes can protect RNA RNases from degradation, so circRNAs are also enriched and stably expressed in exosomes (214). Exosomes derived from cancer cells can target specific organs to promote the formation of pre-metastasis niches (215) and tumor microenvironment (216). Exosomal circRNAs participate in cell proliferation, invasion, EMT, and metastasis through intercellular communication. Shang et al. (217) discovered a new circRNA in colorectal cancer exosomes, circPACRGL, which acts as a sponge for miR-142-3p/miR-506-3p and promotes the expression of transforming growth factor-β1 (TGF-β1). It has been reported that exosomal circSHKBP1 inhibits HSP90 degradation and promotes GC progress through miR-582-3p/HUR/VEGF pathway (134). Recently, Li et al. used circRNA deep sequencing and bioinformatics methods to build a circRNA repertoire, and 3 up-regulated serum exosomal circRNAs (circ0075828, circ0003828, and circ0002976) could be used to screen for high-grade astrocytoma (HGA). Five highly expressed exosomal circRNAs (circ0005019, circ0000880, circ0051680, and circ0006365) were used as HGA prognostic markers. revealed that circular RNAs in HGA exosomes are targets for HGA liquid biopsy and prognostic monitoring (218). At present, progress has been made in the research of exosomal circRNAs, but the mechanism of circRNAs entering exosomes and the role of circRNAs in exosomes are still unclear.

In summary, the prospects of circRNAs as biomarkers for liquid biopsy and therapeutic targets are promising, but there are few studies at present.

## 5 CircRNAs and Future Therapeutic Opportunities

CircRNAs are attractive targets for cancer therapy and offer novel cancer treatment techniques. In this section, we will discuss some future perspectives on the usage of circRNAs in cancer therapy.

As mentioned above, more and more studies have demonstrated that dysregulation of circRNAs in cancer can promote or inhibit cancer (**Table 3**). While the up-or down-regulation of certain circRNAs is linked to clinical aspects such as TNM and other related phases, differentiation, or survival (142, 219). This shows that circRNAs actions are context-dependent, making it difficult to categorize circRNAs as oncogenic or tumor suppressors.

CircRNAs rely on the sponge action of miRNAs to promote cancer progression. In recent years, small molecule inhibitors (SMIs) and small molecule degradants (SMDs) of miRNAs have been reported for drug therapy, so whether it is possible to develop blockers targeting miRNAs to reduce the cancer-promoting activity of circRNAs (220). Of course, the specificity of the drug requires other biotechnological validation and the safety of the drug also needs to be assessed. In addition, when cancer develops, some critical circRNAs are greatly up-regulated. Can it decrease cancers by reducing the number of cancer-promoting circRNAs without influencing the expression of their parental genes? It may be able to regulate the occurrence of back-splicing events by focusing on the splicing mechanisms that affect circRNAs. For example, Tassinari et al. demonstrated that downregulation of the RBP splicing factor ADAR1, which controls circular RNA biogenesis, is sufficient to strongly inhibit glioblastoma growth *in vivo* (221). This inspires the prospect of a technique that modulates RBP to suppress circular RNA expression.

Finally, gene editing techniques such as CRISPR/Cas13 has been applied to RNA editing (222), Whether circRNAs can also be edited to reduce or increase activity. Recently, Ishola et al. found that CRISPR/Cas13a-mediated knockdown of circ0000190 reduced the proliferation and migration of non-small cell lung cancer cells *in vitro* and inhibited tumor growth *in vivo* (223). This also confirms the potential of the novel CRISPR/Cas13a system as a cancer therapy tool.

## 6 Challenges and Perspectives

CircRNAs have been considered splicing errors before, but they have attracted widespread attention in recent years. A lot of innovative research has emerged in the field of circRNAs, but there are still many challenges and problems that need to be solved. From the above-mentioned large number of retrospective reports, it can be seen that the importance of circRNAs is beyond doubt. However, the function of most circRNAs is still unclear, whether there are new undiscovered functions. In addition, the coding potential of circRNAs is often overlooked. And whether the proteins encoded by circRNAs have the functions of conventional proteins. Thousands of circRNAs have been detected, some of them are highly abundant in cancer and some are low in abundance. The detection method for low-abundance circRNAs is not yet mature, and their use as non-invasive biomarkers requires a large number of clinical sample collections. Moreover, their sensitivity and specificity are controversial. In addition, packaging circRNAs into cells to regulate cell activities also requires a lot of research and exploration, so that these studies can truly produce clinical application value. Standardization is needed in many aspects, such as the extraction of differences between detection technologies and the standardization of naming.

In summary, circRNAs play an important role in cancer and provide new insights for cancer management, but the mechanism of action is still in its infancy. The research of circRNAs still has a long way to go.

## Author Contributions

ZL, HQ, and WX contributed to conception and design of this review. YZ wrote the first draft of the manuscript. XZ, YX, and SF wrote sections of the manuscript. All authors contributed to manuscript revision, read, and approved the submitted version.

## Funding

This work was supported by grants from the National Natural Science Foundation of China (no. 81602883), Zhenjiang Key Laboratory of High Technology Research on Exosomes Foundation and Transformation Application (Grant SS2018003), The Foundation for Excellent Young Teachers of Jiangsu University, a project of social development in Zhenjiang (No. SH2021045), and Clinical Medical Science and Technology Development Foundation of Jiangsu University (No. JLY2021013).

## Conflict of Interest

The authors declare that the research was conducted in the absence of any commercial or financial relationships that could be construed as a potential conflict of interest.

## Publisher’s Note

All claims expressed in this article are solely those of the authors and do not necessarily represent those of their affiliated organizations, or those of the publisher, the editors and the reviewers. Any product that may be evaluated in this article, or claim that may be made by its manufacturer, is not guaranteed or endorsed by the publisher.
